# Large volume holographic imaging for biological sample analysis

**DOI:** 10.1117/1.JBO.26.1.016502

**Published:** 2021-01-09

**Authors:** Derk van Grootheest, Temitope Agbana, Jan-Carel Diehl, Angela van Diepen, Vitaly Bezzubik, Gleb Vdovin

**Affiliations:** a Delft University of Technology, Delft Center for Systems and Controls, Delft, The Netherlands; b Delft University of Technology, Design for Sustainability, Delft, The Netherlands; c Leiden University Medical Center, Department of Parasitology, Leiden, The Netherlands; d ITMO University, St Petersburg, Russia

**Keywords:** large volume holographic microscopes, optics, holography, schistosomiasis, diagnosis

## Abstract

**Significance:** Particle field holography is a versatile technique to determine the size and distribution of moving or stationary particles in air or in a liquid without significant disturbance of the sample volume. Although this technique is applied in biological sample analysis, it is limited to small sample volumes, thus increasing the number of measurements per sample. In this work, we characterize the maximum achievable volume limit based on the specification of a given sensor to realize the development of a potentially low-cost, single-shot, large-volume holographic microscope.

**Aim:** We present mathematical formulas that will aid in the design and development and improve the focusing speed for the numerical reconstruction of registered holograms in particle field holographic microscopes. Our proposed methodology has potential application in the detection of *Schistosoma haematobium* eggs in human urine samples.

**Approach:** Using the Fraunhofer holography theory for opaque objects, we derived an exact formula for the maximum diffraction-limited volume for an in-line holographic setup. The proof-of-concept device built based on the derived formulas was experimentally validated with urine spiked with cultured *Schistosoma haematobium* eggs.

**Results:** Results obtained show that for urine spiked with *Schistosoma haematobium* eggs, the volume thickness is limited to several millimeters due to scattering properties of the sample. The distances of the target particles could be estimated directly from the hologram fringes.

**Conclusion:** The methodology proposed will aid in the development of large-volume holographic microscopes.

## 1 Introduction

Particle field holography (PFH) is an important domain developed after the invention of holography by Gabor.^1^ Originating in the early sixties,^2^^,^^3^ it proved a versatile technique to determine the size and distribution of moving or stationary particles in air or in a liquid without significant disturbance of the sample volume. The introduction of solid-state imagers, as replacements of the photographic films, and the recent development of computational methods to numerically reconstruct the object from the recorded hologram, has resulted in the rapid development and application of PFH in diverse fields.^4^^–^^7^

PFH has found significant application in the field of biomedical optics. Several applications exist that are able to analyze larger liquid volumes. The use of holographic techniques combined with flow cytometry for the analysis of liquid samples has been demonstrated.^8^^,^^9^ Furthermore, submerged microscopes have been developed to examine organisms below the water surface.^10^ These interesting techniques, however, suffer from inherent complicated sample handling procedures, which increase the complexity and cost of the system. Holographic microscopes that are able to image a volume of $1.5\;{\mathrm{mm}}^{3}$ have been presented in literature.^11^ Diagnosis of tropical diseases, which requires a larger sample volume, is cumbersome and expensive with the existing techniques. The development of a device that is able to examine liquid samples of around 10 ml with a single-frame would require a sensor with a large sensitive area. To our understanding, a formalism that links the maximum volume that can be examined within a single frame to the sensor specifications and desired resolution does not exist. Therefore, we present a formula for the achievable maximum volume in the case of a collimated in-line setup, a given sensor, and desired resolution when examining opaque spherical or one-dimensional (1D) particles. This formula will aid in the development of a low-cost, single-shot, large-volume holographic microscope.

Reconstructing all relevant particles within a thick volume increases the complexity of focusing. Current techniques either rely on a predetermined set of reconstruction planes to determine the in-focus plane^12^^,^^13^ or an extensive training set for implementation of deep learning.^14^^–^^16^ To circumvent the need for an extensive training set and to reduce the number of reconstruction planes, we propose the use of depth information contained in the digitally registered hologram. This will allow smart numerical reconstruction of target particles at different focal planes throughout the sample.

To verify our methodology, we applied the developed theoretical model to image biological particles, such as *Schistosoma haematobium* eggs, in transparent liquids such as water, phosphate-buffered saline solution (PBS), and urine. *Schistosoma haematobium* eggs are found in urine samples of people infected with urinary schistosomiasis, a neglected tropical disease affecting the lives of more than 250 million people worldwide. According to the World Health Organization (WHO), diagnosis is done using a 10-ml urine sample,^17^ hence the need for an accessible large volume holographic microscope. Previous work, which aimed at combing flow cytometry with digital holography for the detection of *Schistosoma haematobium* eggs, highlighted the limitations of this technique to include (i) accurate control of the sample flow and (ii) long sample processing time due to the sensor size of the scientific camera used.^18^ To complement these gaps, we propose the use of a low-cost consumer camera with larger sensor area and relevant specifications to reduce the complexity and cost of the device. With further optimization, such a device can be developed for quick and affordable detection of *Schistosoma haematobium* eggs in urine samples. The proposed technique, however, can also be applied for (biological) particle analysis in various other liquid samples. We believe the theoretical formula can also be extended to include the effects of refraction to gain a better understanding of the maximum volume when imaging transparent particles.

The theoretical principle and framework are described in Sec. 2. Experimental results are described in Sec. 3; and discussion and conclusion are provided in Sec. 4.

## 2 Theoretical Framework

### 2.1 Fraunhofer Holography

**Fig. 1:**
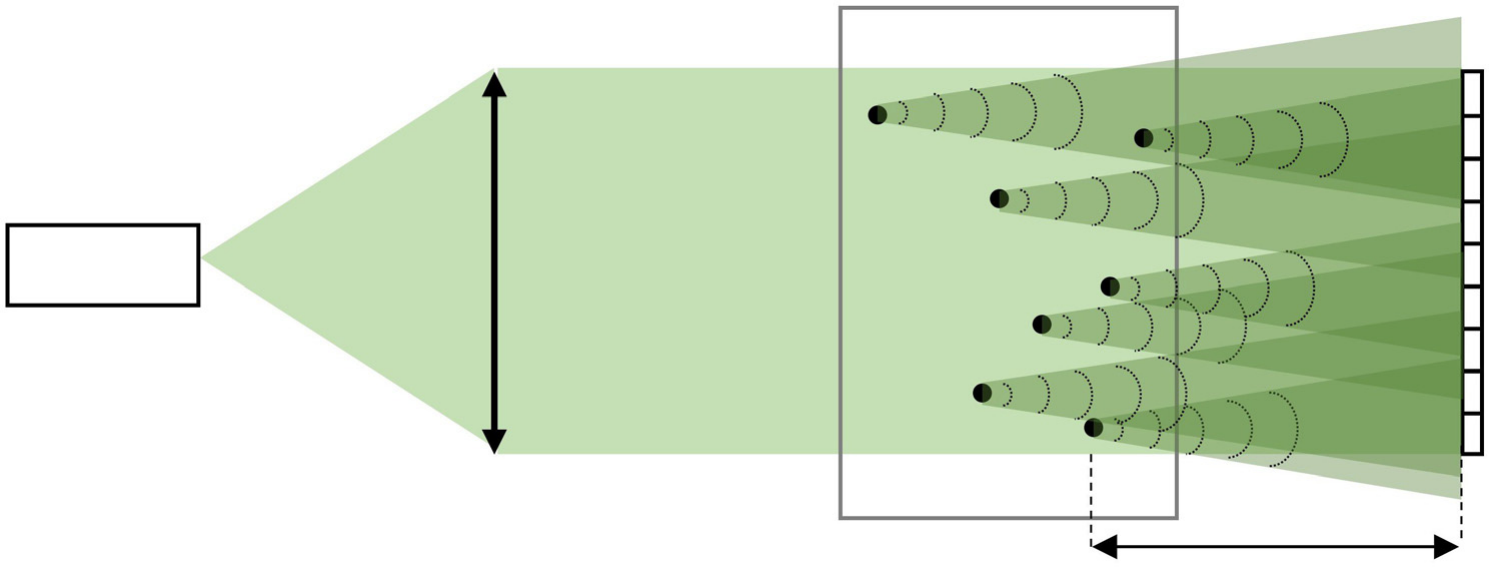
In-line holographic setup with collimated illumination used throughout this article. z is the distance from the object to the sensor.

Fraunhofer (or far-field) holography is the recording of the interference between an object’s far-field diffraction pattern and the coherent quasimonochromatic background illumination as shown in Fig. 1. Strictly speaking, a far-field diffraction pattern is formed when the point of observation and the source are infinitely distant from the illuminated object.^19^ An approximation to this infinite distance is estimated using the so-called far-field condition and it is mathematically expressed as^20^

$$z\gg d^{2}/\lambda =\delta ,$$ (1)

where z is the distance from the object to the hologram, d is the characteristic length of the object, λ is the wavelength of the source, and δ is one far-field distance. In practice, the far-field condition is already met with z=δ.^21^ We have adopted the Fraunhofer approach as the basis for our work because of the simplicity of its analytical formulas. For an opaque object with a circular or spherical geometry described by $D(x_{O})$ illuminated with a coherent quasimonochromatic plane wave, the Fraunhofer pattern is represented as^22^

$$I_{\mathrm{hol}}(x)=1-\frac{k}{\pi z}\overset{˜}{D}(\frac{x}{\lambda z})\sin(\frac{k{\left|x\right|}^{2}}{2z})+\frac{k^{2}}{4\pi^{2}z^{2}}{\left[\overset{˜}{D}(\frac{x}{\lambda z})\right]}^{2},$$ (2)

where $\overset{˜}{D}$ is the Fourier transform of the object, x is the position vector at the recording plane, and k=2π/λ is the wave number. The last term in Eq. (2) is small compared to the other two terms due to the $1/z^{2}$ term and can, therefore, be neglected for a large z. According to Thompson et al. the effect of the twin image, which is the common source of noise in an in-line holographic microscope, is insignificant in the reconstruction of the object when the far-field condition is satisfied.^19^ Furthermore, Eq. (2) also shows that the distance z can be determined directly from the sine term, regardless of the object’s geometry. This can be used to effectively determine the plane of focus of each particle in a volume and decrease the number of reconstruction planes needed to determine the focus plane. Considering these features makes in-line Fraunhofer PFH a promising method for analyzing a large volume of microparticles.

#### 2.1.1 Opaque vs. transparent particles

As mentioned, we assume the particles to be opaque. Many biological samples, however, are (semi)-transparent. Limiting the theory to opaque particles reduces the scattering to only include diffraction. When the particles are (semi)-transparent, refraction also has to be taken into account. This requires knowledge of the relative refractive index of the particles, which is not always known. Analytical formulas for transparent particles are given in literature and their effects have been observed in practice.^23^^–^^25^ We hope the formalism we present in this work will be extended (using the aforementioned literature) to take into account the effects of refraction in the near future. Opaque particles and objects are the focus of the work presented in this article.

### 2.2 Diffraction-Limited Resolution

The maximum volume in which the target objects can be reconstructed first of all depends on the resolution R needed for accurate reconstruction. Several factors limit the resolution throughout the sensor’s volumetric field of view. The first is the sensor’s role as an aperture resulting in a diffraction-limited resolution. This limit is shape-dependent. Biological particles can be roughly grouped into spherical and line objects. The following approach is done for an opaque sphere and later generalized for opaque line objects. The diffraction-limited resolution can be determined from the intensity distribution of an opaque sphere located at a distance z

$$I_{\text{sphere}}(r)=1-\frac{kd^{2}}{4z}\;\sin(kr^{2}/2z)[\frac{2J_{1}(kdr/2z)}{kdr/2z}],$$ (3)

where r is the radial distance from the center of the hologram, and d is the diameter of the sphere. Equation (3) has been derived by inserting the Fourier transform of an opaque object into Eq. (2) and taking other assumptions stated earlier into consideration.

**Fig. 2:**
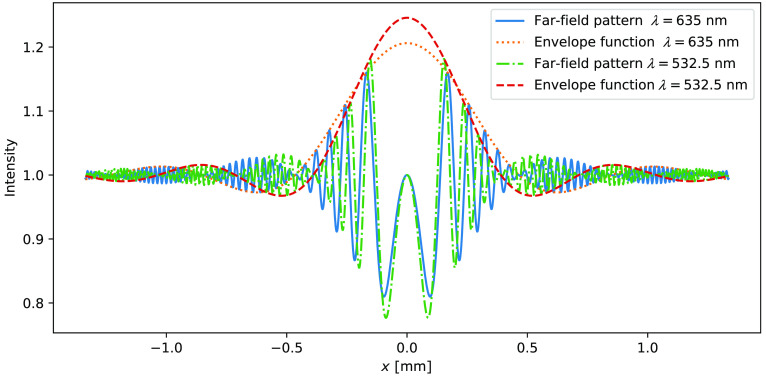
The far-field intensity distribution given by Eq. (3) for a sphere with d=50  μm, z=30  mm, and two wavelengths λ=635  nm and 532.5 nm. x is the distance along the recording plane, r=|x|. The dashed curve is the envelope function given by setting the sine term in Eqs. (3) to (1).

Figure 2 shows two plots of Eq. (3) and the corresponding envelope functions for a sphere with d=50  μm and z=30  mm, illuminated with two different wavelengths. The envelope function is obtained when the faster oscillating sine term in Eq. (3) is neglected. The central peak of the envelope function needs to be recorded to reconstruct the sphere. The first zero of the first-order Bessel function kdr/2z=3.8317, provides the radius $r_{0}$ of this central peak

$$r_{0}=\frac{3.8317\cdot 2z}{kd}$$ (4)

Diffraction-limited resolution is reached when $r_{0}=\frac{w}{2}$, where w is the width of the sensor. If $r_{0}>\frac{w}{2}$, part of the central peak is not recorded on the sensor making correct numerical reconstruction impossible. Defining the resolution as the radius of the smallest resolvable sphere ($R=\frac{d}{2}$), the diffraction-limited resolution is then given as

$$R_{\mathrm{diff}}=\frac{1.22\lambda z}{w}$$ (5)

This is the case for a particle that lies on the optical axis. When a particle is shifted in-plane away from the optical axis, the intensity pattern shifts accordingly and part of the central peak is not recorded by the sensor. For a particle lying at a distance Δr to the optical axis, the diffraction-limited resolution is given as

$$R_{\mathrm{diff}}=\frac{1.22\lambda z}{w-2\Delta r}$$ (6)

### 2.3 Pixel Pitch Limited Resolution

The second limitation on the resolution is the resolvability of the recording medium. In the case of a sensor, this is the sensor’s pixel pitch p. For accurate estimation of the distance z from the sine term in Eq. (2), these finer fringes need to be sampled at a sampling rate of at least twice the Nyquist frequency. The difference between the n’th and the (n+1)’th fringe being 2π gives

$$r_{n+1}^{2}-r_{n}^{2}\approx 2r_{n}\Delta r_{n}=\frac{4\pi z}{k}$$ (7)

where $\Delta r_{n}=r_{n+1}-r_{n}$ and $\Delta r_{n}\ll r_{n}$. Substituting $r_{0}$, the radius of the central peak from Eq. (4), for $r_{n}$ in Eq. (7) provides an expression for the spacing $\Delta r_{n}$ between the outer fringes at the edge of the central peak of the envelope function

$$\Delta r_{0}=\frac{2\pi z}{kr_{0}}=\frac{2\pi zkd}{k\cdot 3.8317\cdot 2z}=\frac{d}{1.22}$$ (8)

It is worth noting that the index 0 in $r_{0}$ is the index for the central peak of the envelope function and not the index for the finer fringes. To accurately sample the whole central peak, according to the Nyquist criterion, the sampling rate has to be at least $2/\Delta r_{0}$. With a sampling rate of 1/p this results in the resolution being bounded by the pixel pitch as

$$R_{p}=1.22p$$ (9)

### 2.4 Signal-to-Noise-Limited Resolution

A third limitation on the resolution is due to the signal-to-noise ratio (SNR), which depends on the particle density in the volume, the thickness of the sample volume, movement of particles, and the detectability of the interference fringes at the sensor. Detectability in this case implies that the amplitude modulations of the individual patterns, belonging to the scattering of a particle, exceeds the noise level. For bit depths >4  bits, it has been shown that the image reconstruction is not influenced substantially by the recording of the fringes.^26^

Movement of particles causes smearing of the fringes,^27^^,^^28^ and therefore, contributes to the SNR. The general rule is that a particle may translate in-plane a 10’th of its diameter during the exposure time.^29^ If this requirement is met, any movement in the longitudinal direction has no significant effect.

Generally, the aforementioned requirements are met in practice and then the main source governing the SNR is the shadow density.^30^^,^^31^ High density of particles will cause speckle patterns, which makes the reconstruction of the individual objects practically impossible. The speckles are due to the complex scattering inside the sample. A general rule is that at least 80% of the light should not scatter off the particles.^32^

The sensor acts as an aperture limiting the resolution as shown in Eq. (5). In the case of SNR-limited resolution, the width of the sensor w in Eq. (5) should be replaced with the SNR diameter $w_{\mathrm{SNR}}$. This diameter is determined as the diameter of the sensor area for which the intensity modulations exceeds the noise level. This gives

$$R_{\mathrm{SNR}}=\frac{1.22\lambda z}{w_{\mathrm{SNR}}}$$ (10)

where $w_{\mathrm{SNR}}$ is always ≤w, increasing the resolution. A lower SNR reduces the aperture of the system.

### 2.5 Maximum Volumetric Field of View

Combining the equations for the diffraction-limited resolution with the far-field condition, we get two bounds on the distance z within which the Fraunhofer PFH equations are applicable and the desired resolution throughout the volume is met

$$\frac{d^{2}}{\lambda }<z<\frac{Rw^{\prime }}{1.22\lambda }$$ (11)

where $R\geq R_{p}$ is the desired resolution and $w^{\prime }$ is either $w_{\mathrm{SNR}}$ in the case of a SNR-limited resolution or w−2Δr in the case of diffraction-limited resolution. d is the characteristic object dimension is taken as the diameter of the sphere. As the lower bound depends on the size of the object, it should be noted that when examining a sample containing a wide range of particle sizes the size of the largest particle present should be taken to determine the lower bound. In this way, the far-field condition is met for the whole volume. However, it may be that this lower bound is larger than the upper bound considering the desired resolution or noise present. Considering the fact that the lower bound of z scales with $d^{2}$, this theory is best used in samples containing a small range of particle sizes. Having a large range of particle sizes may cause the fringes of the smaller particles to get lost in the noise level due to the weak signal caused by the relatively large distance to the sensor.

#### 2.5.1 Opaque line objects

In the case of opaque line objects, the presented approach still holds apart from some slight changes. The intensity distribution of an opaque line is given as^33^

$$I_{\text{line}}(x)=1-2d\sqrt{\frac{1}{\lambda z}}\;\cos(\frac{kx^{2}}{2z}-\frac{\pi }{4})[\frac{\sin(kdx/2z)}{kdx/2z}]$$ (12)

where d is the width of the line. Line objects have a higher fringe visibility as compared to spherical objects.^34^ This results in a lower SNR-limited resolution as compared to spherical objects. In fact, all limits change slightly due to the fact that the first zero from sin(kdx/2z) occurs at π as opposed to the 3.8317 from the Bessel function. This results in the resolution for line objects and spherical objects to relate as $R_{\text{sphere}}=1.22R_{\text{line}}$. The estimation of z is now done using the cosine term in Eq. (12).

#### 2.5.2 Maximum volumetric field of view

When the parameters of the source and sensor are known, the maximum volume for diffraction-limited resolution can be determined. The maximum volume V is given as

$$V=\frac{\pi {\left(Rw-Sd^{2}\right)}^{3}+6R(h-w){\left(Rw-Sd^{2}\right)}^{2}}{12S\lambda R^{2}}$$ (13)

where h×w is the dimension of the sensor, R is the desired spatial resolution, S is the shape parameter, which is 1 for line objects and 1.22 for spherical objects, d is the diameter in the case of spherical particles and the width in the case of line objects, and λ is the wavelength of the source. Equation (13) is derived using the lower and upper bounds for z from Eq. (11) with special attention to the fact that the diffraction pattern spreads out conically in space. For the full derivation of the equation, the interested reader can please refer to the Appendix. In the case of SNR-limited resolution, no analytical formula exists due to $w_{\mathrm{SNR}}$ varying locally over the sensor. However, in the case of SNR-limited resolution, the volume will not exceed the volume given by Eq. (13).

### 2.6 Determining Object—Sensor Distance

To determine the distance z to the particle directly from the hologram data, the maxima and minima of the fringes are measured and linked to the maxima and minima of the sine in the case of spherical particles, and the cosine in the case of line objects. This results in the following estimation for z in the case of spherical particles

$$z=\frac{r_{n}^{2}}{\lambda (n-\frac{1}{2})}$$ (14)

and in the case of line objects

$$z=\frac{r_{n}^{2}}{\lambda (n-\frac{3}{4})}$$ (15)

where n is the index of the minima and maxima as seen from the center of the hologram and $r_{n}$ is the radial distance from the center of the hologram fringes corresponding to the n’th minima or maxima.

## 3 Results

### 3.1 Experimental Validation

**Fig. 3:**
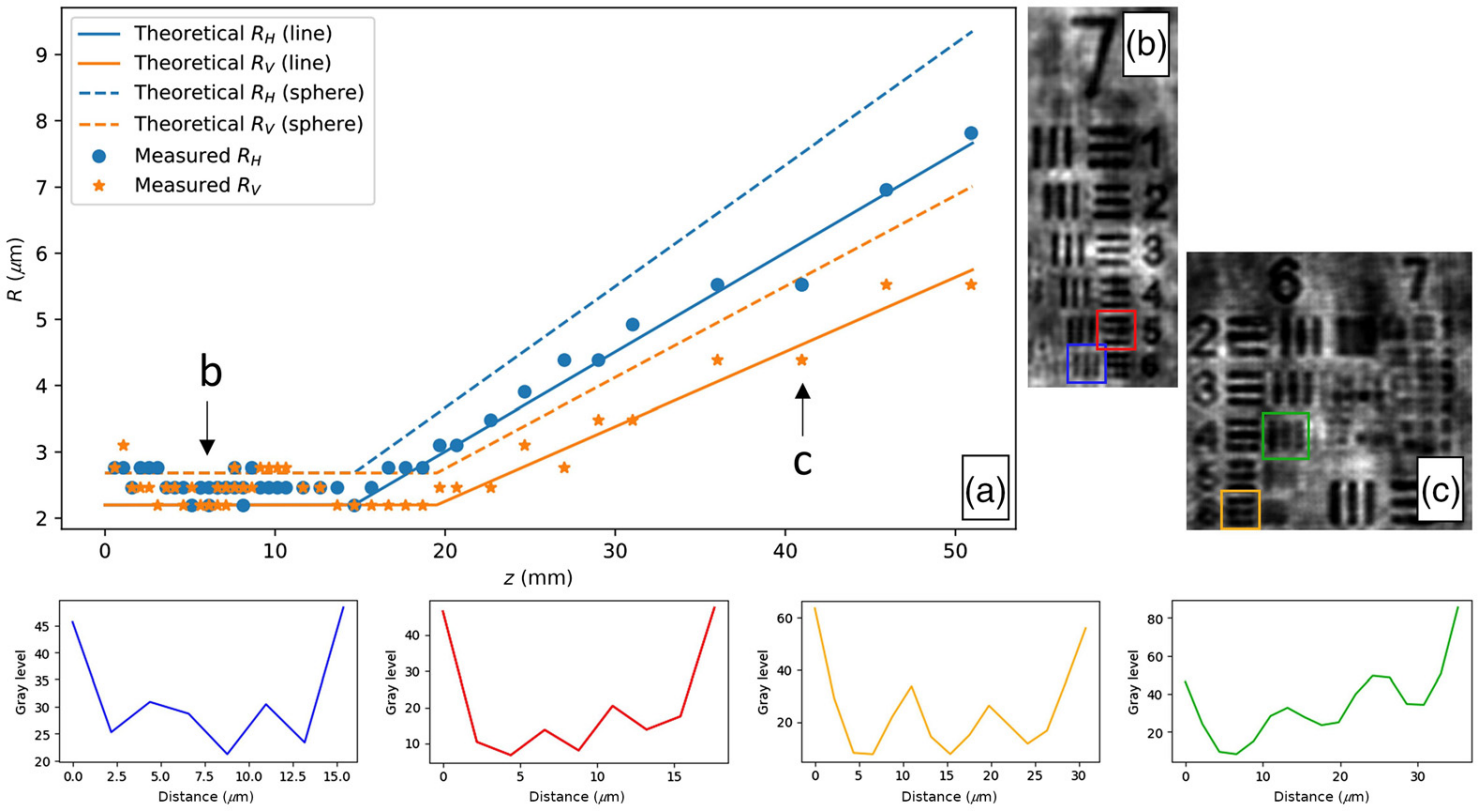
(a) Resolution R for objects at different distances z. Due to the rectangular shape of the sensor, the resolution is split into the horizontal ($R_{H}$, marked in blue) and vertical ($R_{V}$, marked in orange) resolution. The theoretical resolution bounded by the sensor dimensions [Eq. (5)] and pixel pitch [Eq. (9)] is plotted for both spherical particles (dashed lines) and line objects (solid lines). The dots and stars mark the measured horizontal and vertical resolution using a USAF resolution chart. (b) and (c) Reconstruction of the USAF resolution chart positioned at a distance z=7.12  mm in (b) and at z=40.97  mm in (c). The four graphs at the bottom mark the intensity profiles of the smallest resolved elements, marked with squares in corresponding colors in (b) and (c).

To see the effects of the sensor size and pixel pitch on the systems spatial resolution, a United States Air Force (USAF) resolution chart was imaged over several distances z along the optical axis. The USAF resolution chart consists of sets of horizontal and vertical bars. The resolution we use is the half-pitch resolution, defined as the width of a single bar from the smallest discernible element on the USAF chart. For the experiment, we used a 12-bit iDS UI-3482LE-M CMOS monochrome camera with a pixel pitch of 2.2  μm and a sensor size of 2560×1920  pixels configured to operate in portrait mode. A Thorlabs HLS635 light source with a central wavelength of 635 nm was used to illuminate the chart with collimated illumination as shown in Fig. 1. Using the angular spectrum method^35^ as described by Latychevskaia and Fink,^36^ the resolution chart was reconstructed for each distance. We are aware that to prevent aliasing at larger distances, zero-padding should be applied.^37^ However, upon reconstruction, the only artifacts observed were present at the edges of the reconstruction, whereas the central part, which corresponds to the smaller elements, showed no aliasing. Therefore, zero-padding was not used. The measured results are shown in Fig. 3(a). As a result of the rectangular shape of the sensor, a difference in resolution was observed depending on the orientation of the bars in the chart. In our observation, the vertically oriented bars had worse resolutions for large z as compared to the horizontal bars. This is due to the sensor operation in a portrait mode. The sensor in this mode has a shorter width in comparison to the height. Figure 3(a) also shows the theoretically predicted resolution as given by Eqs. (5) and (9) for spherical particles (plotted in dashed lines) and the corresponding for line objects (plotted in solid lines) plotted alongside the measurement results. It can be seen that the measurement results follow the theoretical curve for 1D line objects, which is expected considering the chart consists of bars.

In the region where the resolution is bounded by the pixel pitch, we observed that the measured resolution is often worse than the theoretical resolution. The deterioration in resolution can be attributed to the twin image from the larger elements on the resolution chart not being suppressed. Because these larger elements do not generate a far-field hologram as opposed to the smaller elements, the twin image is more present upon reconstruction, worsening the resolution. When examining a sample consisting of objects within the same order of magnitude, the volume can be placed at such a distance that the twin-image effects can be suppressed for all objects.

Determining which element is still visible is open to some ambiguity, and this explains why some measurement results shown on the plot are below the theoretical resolution as is the case for z=40.97  mm. Figures 3(b) and 3(c) show the reconstruction of the USAF resolution chart positioned at a distance z=7.12  mm and at z=40.97  mm, respectively. For better visualization of the smallest elements, the reconstruction of the hologram was interpolated with a sine function to provide the optimal interpolation for band-limited signals.^38^ Although no new information is added, the reconstructed image quality is improved. The smaller elements that are not reconstructed due to sensor limitations contribute to the signal making it essentially not band limited. However, the contribution of these smaller elements is so small that the signal can be approximated as a band-limited signal. Figure 3(c) clearly shows the difference in resolution for the vertical and horizontal bars, as mentioned. The four colored graphs in Fig. 3 show the intensity plot of the smallest resolvable elements in Figs. 3(b) and 3(c) without applying any sine interpolation.

### 3.2 Biological Samples

#### 3.2.1 From sphere to egg

Several biological samples were imaged to demonstrate the practical application of the theoretical principle. Since biological samples are hardly ever pure spheres or 1D line objects, we first had to prove that the framework would hold for different shapes. The micro-sized objects under study are *Schistosoma haematobium* eggs. *Schistosoma haematobium* eggs have a size that ranges between 110 and 170  μm in length and 40 to 70  μm in width at full maturity and can be smaller in premature stages.^39^^,^^40^ The eggs contain a terminal spine of size ∼10  μm, which is a typical characteristic of the species. Using data from near-field experiments,^18^ we numerically extrapolated the data to the far-field and found that the fringe pattern of a sphere with a diameter equal to the width of the egg showed good correspondence with the far-field fringe pattern of the egg. Therefore, the width of the egg is chosen as the characteristic object dimension d in Eq. (11). This means that the sensor needs to be placed at a distance of 3 to 9.2 mm from the nearest egg, depending on the egg size.

#### 3.2.2 Experimental results

**Fig. 4:**
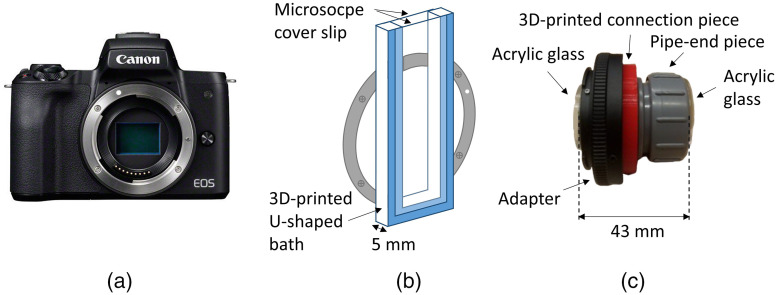
(a) Canon EOS M50^41^ containing a 24MP APS-C CMOS color sensor. (b) Three-dimensional (3D) printed U-shaped bath with a volume depth of 5 mm. (c) Polyvinyl chloride pipe-end piece with acrylic glass slides on both ends connected to an adapter for mounting on the Canon EOS M50.

**Fig. 5:**
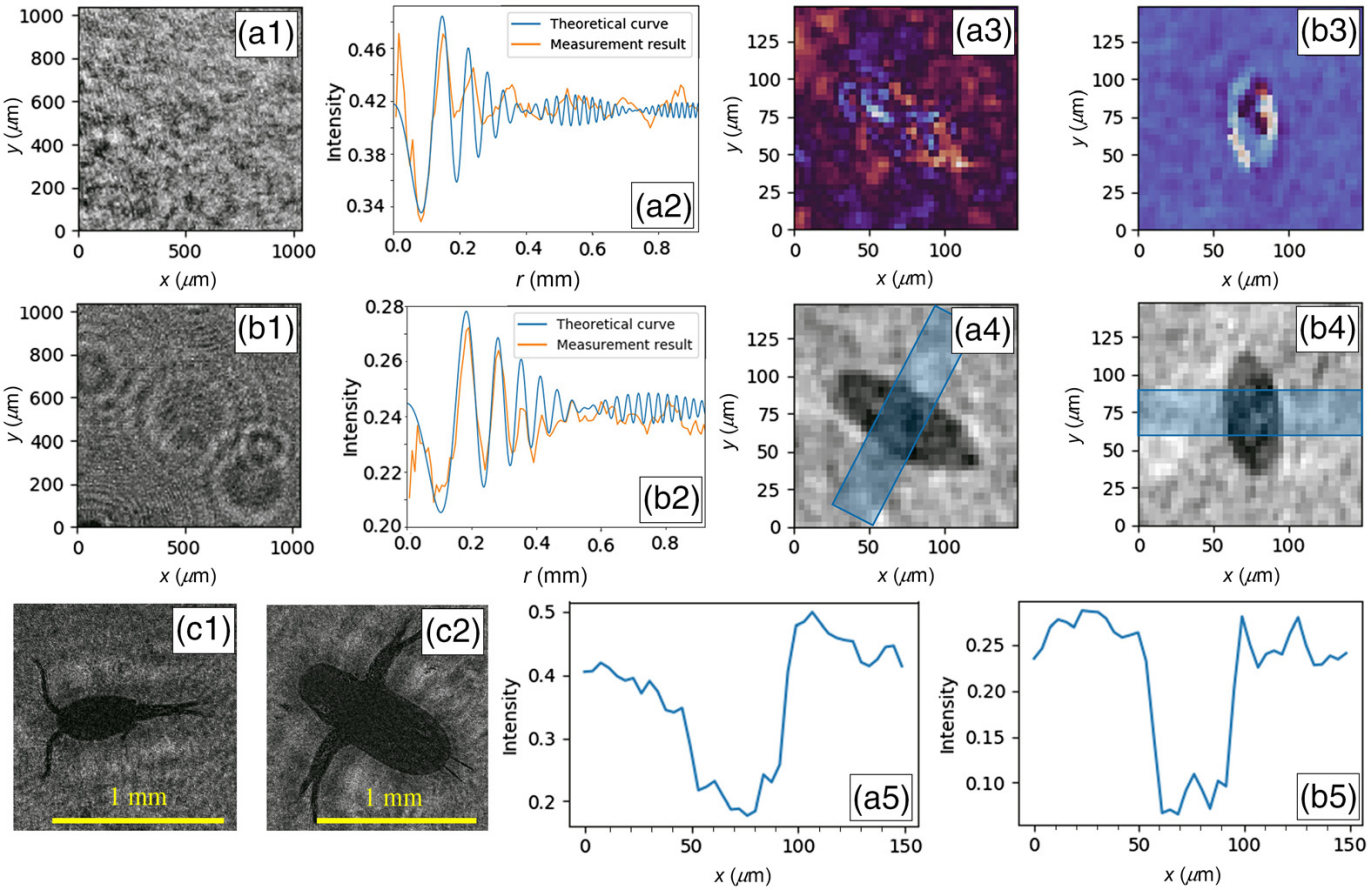
Measurement results for urine (a), PBS solution (b) and water (c). (a1) Hologram section belonging to a *Schistosoma heamatobium* egg in a 5-mm-thick volume filled with urine. (a2) Normalized average intensity of consecutive rings around the location of the egg in a1 compared to the analytical function for a sphere with the diameter equal to the width of the egg. (a3) Phase reconstruction of a1. (a4) Intensity reconstruction of a1. (a5) Average intensity profile of the blue rectangular area in a4. (b1) Hologram section belonging to a *Schistosoma heamatobium* egg in a 43-mm-thick volume filled with PBS solution. (b2) Normalized average intensity of consecutive rings around the location of the egg in b1 compared to the analytical function for a sphere with the diameter equal to the width of the egg. (b3) Phase reconstruction of b1. (b4) Intensity reconstruction of b1. (b5) Average intensity profile of the blue rectangular area in b4. (c1) and (c2) Two small water bugs imaged in water contained in a 43-mm-thick volume.

Using the commercially available^41^ 24MP APS-C CMOS color sensor with pixel pitch p=3.71  μm and sensor dimensions of 6012×4008  pixels and a light source with a central wavelength of 532.5 nm, we conducted several experiments. Results obtained are shown in Fig. 5. The containers used to hold the samples are shown in Fig. 4.

In the first experiment, a 5-mm-thick volume [Fig. 4(b)] of urine spiked with *Schistosoma. haematobium* eggs was used. The volume was heavily spiked to ensure easy detection of eggs in a registered image. The sensor was placed at a distance of ∼25  mm to the urine to ensure the far-field condition applied to all egg sizes. Figure 5(a4) shows a reconstructed intensity image of an egg at a distance of z=27.65  mm to the sensor. The terminal spine can also be observed in the image. The corresponding hologram fringes are shown in the noisy image in Fig. 5(a1). When taking the average normalized value of concentric rings around the center of the egg, we see that the first two oscillations match the theoretical curve for a spherical particle positioned at the same distance with a diameter equal to the width of the egg [Fig. 5(a2)]. The theoretical curve is Eq. (3) multiplied with a scale factor to have the same background intensity as the measurement data. To determine the hologram quality we calculated the fringe contrast^42^

$$C=\frac{I_{\max}-I_{\min}}{I_{\max}+I_{\min}}$$ (16)

where $I_{\max}$ is the maximum fringe intensity and $I_{\min}$ the minimum fringe intensity. Due to the concentric nature of the fringes, $I_{\max}$ and $I_{\min}$ are taken from the measurement results in Fig. 5(a2). The calculated value is C=0.18. For expert diagnosis, the shape, size, and observation of the terminal spine are most important for a diagnostic conclusion. The phase reconstruction image shown in Fig. 5(a3) did not add any useful additional information. With the volume thickness extended beyond 5 mm, using the container shown in Fig. 4(c), the quality of the reconstruction was too poor, making the terminal spine of the egg less visible in the image. The sample volume was placed at a distance of ∼9  mm to the sample placing the sample within the bounds stated by Eq. (11), with $w^{\prime }=w$. From this we conclude that for imaging *Schistosoma haematobium* eggs in urine, the volume is strongly bounded due to scattering in the volume.

We replaced the urine by a PBS solution to see the scattering effects. PBS has minimal contamination; this makes it a more optically transparent liquid compared to urine. Figure 5(b4) shows an intensity reconstruction of a small premature egg lying at a distance z=42.70  mm in a 43-mm-thick volume. The corresponding hologram fringes shown in Fig. 5(b1) clearly shows less noise and improved fringe visibility. In fact, the frequency of the first six oscillations tends to match the theoretical curve, as can be seen in Fig. 5(b2). However, the fringe contrast is lower with a value of C=0.13. This lower value is due to the fact that the egg lies further away^33^ and the mean recorded intensity is lower than the case with urine.

A third test was done with samples of water randomly collected from a water pond close to the laboratory. The aim of the experiment was to optically screen the water sample and analyze the particles present. The water was transferred into a 43-mm-thick volume and the hologram was registered accordingly. Interestingly, alongside other particles, two bugs were observed in the reconstructed holographic image, as shown in Figs. 5(c1) and 5(c2). The body of the bugs results in a near-field hologram, which explains the halo around the reconstruction, but the antlers having a width of around 10  μm are recorded as a far-field hologram. The obtained experimental result confirms the potential application of this technique to other fields of science.

#### 3.2.3 Estimating distance

**Fig. 6:**
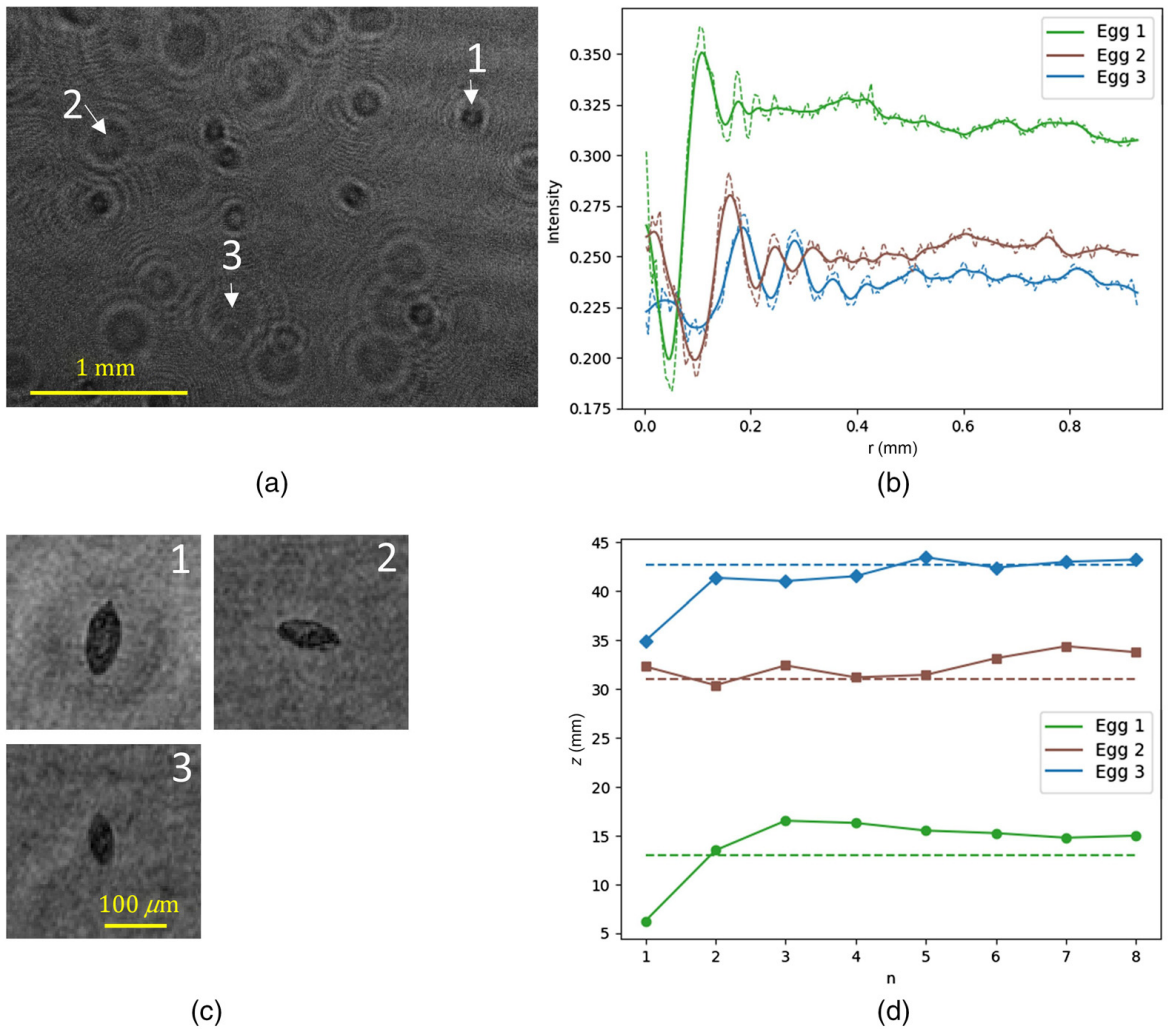
(a) Hologram with three labeled eggs in PBS solution; (b) average intensity of consecutive rings in dashed lines and a Gaussian smoothed curve in solid; (c) reconstruction of the three labeled eggs; (d) estimation of z using the position of the smoothed extrema in (b) (the horizontal dashed lines mark the manually determined distance z).

**Fig. 7:**
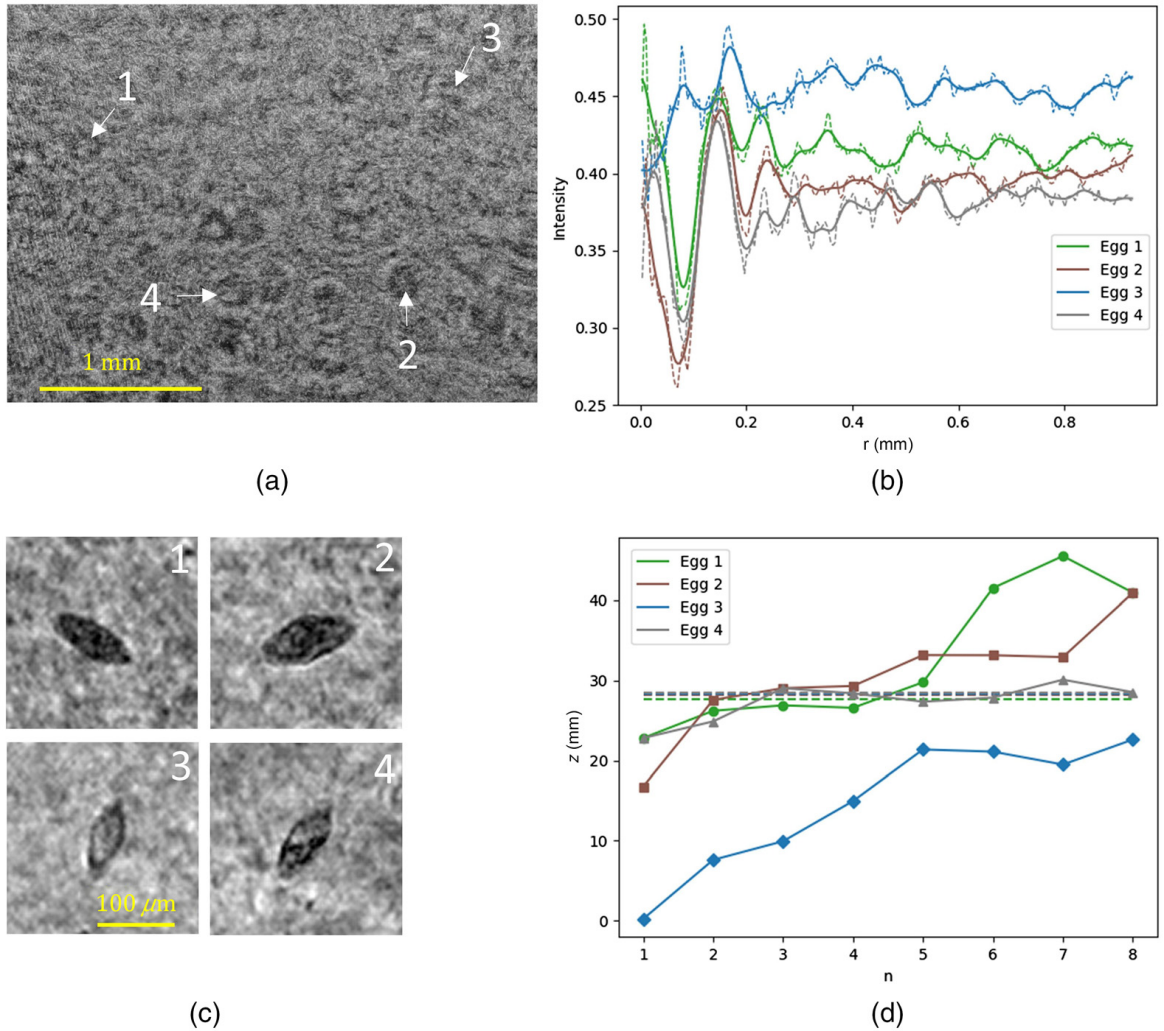
(a) Hologram with four labeled eggs in urine; (b) average intensity of consecutive rings in dashed lines and a Gaussian smoothed curve in solid; (c) reconstruction of the four labeled eggs; (d) estimation of z using the position of the smoothed extrema in (b) (the horizontal dashed lines mark the manually determined distance z).

The distance to the sensor was estimated for three eggs in a PBS solution (Fig. 6) and four eggs in urine (Fig. 7), using Eq. (14). The intensity profile, acquired by taking the average intensity of consecutive rings, was smoothed before determining the location of the minima and maxima, as shown in Figs. 6(b) and 7(b). Figures 6(c) and 7(c) show the reconstructions of the eggs. The estimate for z, based on the location of the minima and maxima, is plotted alongside the manually determined focus distance in Figs. 6(d) and 7(d). Comparing Figs. 6 and 7, we see that the number of usable extrema is lower in the case of urine. This is due to the lower SNR causing the extrema associated with the higher n values to be lost in the background noise. From Fig. 6(b) and from the calculated fringe contrasts of 0.33, 0.21, and 0.13 for egg 1, 2, and 3, respectively, we can see that the fringe contrast drops as the eggs are further displaced. Figures 6(d) and 7(d) show that it is possible to reduce the region within which frames need to be reconstructed to find the plane of focus. It is good to note that this region is around 3- to 5-mm wide, which means that it is not yet practical to use it with samples of comparable thickness such as the 5-mm urine sample. As the estimation of z scales with $r_{n}^{2}$ a slightly different value of $r_{n}$ already causes a significant shift in z

$$\Delta z=\frac{{\left(r_{n}+\Delta r\right)}^{2}-r_{n}^{2}}{\lambda (n-\frac{1}{2})}\approx \frac{2\Delta rr_{n}}{\lambda (n-\frac{1}{2})}$$ (17)

For the extrema n=2 from egg 2 in Fig. 7, a shift of Δz=2  mm is caused by a shift Δr≈5  μm. With a pixel pitch of 3.71  μm, this means that the smoothing of the intensity curve has to be done with great care for the depth estimation to be of use in thinner samples. For thicker samples, the benefit is clear. It is important to note that there is a tendency to overestimate the focus distance and this could be due to the fact that the eggs are not opaque, so refraction plays a role. The fact that eggs are not perfectly spherical could also be a challenge. Egg 3 in Fig. 7 is clearly semitransparent and shows underestimation of the distance. This most likely is due to the noisy fringe pattern.

#### 3.2.4 Color camera

The 24MP APS-C CMOS color sensor used throughout the experiments with the biological samples is present in the CANON EOS M50.^41^ The sensor was specifically selected because of its large sensor area (which translates to wide field of view), its pixel pitch, and its low-cost (being five times cheaper than comparable monochrome cameras). Recording of monochromatic light with this color sensor was not a major challenge in our case. The internal debayering algorithm provided an accurate monochromatic hologram, which produced a reconstructed image with a similar resolution as would have been obtained with a monochromatic sensor of same pixel pitch. This shows the prospects of implementing affordable consumer sensors in the holographic imaging of biological samples.

## 4 Discussion and Conclusion

We have presented a formalism defining the maximum imaging volume for particles suspended in clear liquid with any given sensor and the desired resolution in a collimated in-line setup. From the theoretical analysis and experimental results, we established that the maximum volume of clear liquid that can be analyzed largely depends on the size of the sensor, the size of the particle, the specific wavelength, with a shorter wavelength providing better spatial resolution, the shape of the particles (1D particles/objects are resolved at improved spatial resolution), and the SNR of the holographic image.

We believe the scattering in the sample to be the main factor limiting the volume of single-shot imaging of the biological samples. Reduced fringe visibility due to scattering results in loss of resolution. In our experiments, scattering has been limiting the volume of urine samples spiked with *Schistosoma haematobium* eggs. Scattering increased with an increase in the thickness of the urine layer. We observed a maximum analyzable thickness of 5 mm. Replacing the urine with clear PBS solution spiked with eggs of *Schistosoma haematobium*, we were able to obtain clear single-shot images in a whole WHO-recommended 10-ml sample, with a thickness of up to 43 mm.

We have shown that the distance to each particle can be estimated from simple analysis of the fringe pattern. Thick samples, to a large extent, reduce the number of reconstruction planes needed to determine the plane of focus of the particle, thus reducing the processing times for thick samples with numerous particles at different distances. As the estimation is very sensitive to the measured position of extrema in the fringe pattern, improving the determination of the extrema locations is the first step toward fast focusing.

Although the model of opaque particle has produced a good match to the experimental measurements, it would be useful to expand the formalism to encompass the effects of refraction to make the formalism applicable to a broader range of (semi-) transparent biological objects. Also, as scattering remains the main limiting factor, research into the effects of particle density on the axial position estimation should be conducted to verify the applicability of our proposed technique for varying sample densities.

To verify our developed model, we implemented a simple in-line holographic microscope, using a Canon M50 mirrorless camera as a sensor. The experimental results obtained with biological samples have shown promising results applicable to various domains of volumetric particle analysis in the lab and in the field.

## 5 Appendix

**Fig. 8:**
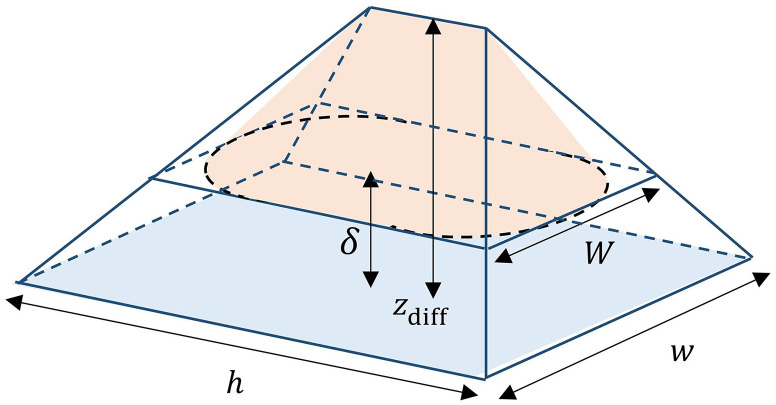
The maximum volume as described by Eq. (13). h and w are the sensor dimensions, δ is the one far-field distance, $z_{\mathrm{diff}}$ is the maximum distance according to the diffraction limited resolution [Eq. (5)].

Equation (13) equals the orange prism-shaped volume in Fig. 8. The orange prism is bounded by one far-field δ and the maximum distance according to the diffraction $z_{\mathrm{diff}}$. The diffracted pattern spreads out conically in the case of a spherical particle and symmetrically in the case of line objects. Taking all possible orientations of line objects into account also results in the the conical shape of V. To calculate the volume, the volume can be split into a conical segment and a triangular prism segment

$$V=\frac{\pi W^{2}(z_{\mathrm{diff}}-\delta )}{12}+\frac{1}{2}(h-w)W(z_{\mathrm{diff}}-\delta );$$ (18)

using similar triangles, W is expressed as

$$W=\frac{w(z_{\mathrm{diff}}-\delta )}{z_{\mathrm{diff}}}$$ (19)

and using Eq. (11), $\left(z_{\mathrm{diff}}-\delta \right)$ can be expressed as

$$(z_{\mathrm{diff}}-\delta )=\frac{Rw-Sd^{2}}{S\lambda }.$$ (20)

Plugging Eqs. (19) and (20) into Eq. (18) gives Eq. (13)

$$V=\frac{\pi {\left(Rw-Sd^{2}\right)}^{3}+6R(h-w){\left(Rw-Sd^{2}\right)}^{2}}{12S\lambda R^{2}}.$$ (21)
